# How much difference does DSM-5 make? A longitudinal evaluation of DSM-IV and DSM-5 eating disorders in a population-based cohort of adolescents

**DOI:** 10.1186/2050-2974-1-S1-O64

**Published:** 2013-11-14

**Authors:** Karina Allen, Susan Byrne, Wendy Oddy, Ross Crosby

**Affiliations:** 1 Centre for Child Health Research, Telethon Institute for Child Health Research, Australia; 2 School of Psychology, The University of Western Australia, Australia; 3 Department of Clinical Neuroscience, University of North Dakota School of Medicine and Health Sciences, USA; 4 Department of Biostatistics, Neuropsychiatric Research Institute, USA

## Aims

This study aimed to provide data on the relative prevalence of DSM-IV and DSM-5 eating disorders at three time points in adolescence, for males and females. It also aimed to examine associations between DSM-IV and DSM-5 eating disorders and depressive symptoms and quality of life.

## Methods

Participants (N=1,383; 49% male) were drawn from the Western Australian Pregnancy Cohort (Raine) Study, a prospective, population-based cohort study that has followed participants from pre-birth to young adulthood. Changes in eating disorder prevalence over time were considered using generalised estimating equations.

## Results

Eating disorder prevalence rates increased significantly from age 14 to age 20, irrespective of the diagnostic system used. The prevalence of DSM-5 eating disorders was significantly higher than the prevalence of DSM-IV eating disorders for females, but not for males. 'Unspecified' eating disorders were significantly less common when applying DSM-5 than DSM-IV criteria, for males and females, but still accounted for 15% to 40% of the DSM-5 cases. All eating disorder diagnoses were associated with depressive symptoms and poor mental health quality of life.

## Conclusions

Results provide further support for the clinical utility of DSM-5 eating disorder criteria, and for the significance of binge eating disorder and 'not elsewhere classified' disorders.

This abstract was presented in the **Understanding and Treating Eating Pathology** stream of the 2013 ANZAED Conference.

